# Earthquakes Have Accelerated the Carbon Dioxide Emission Rate of Soils on the Qinghai‐Tibet Plateau

**DOI:** 10.1111/gcb.70024

**Published:** 2025-01-10

**Authors:** Peijun Shi, Xiaokang Hu, Heyi Yang, Lu Jiang, Yonggui Ma, Haiping Tang, Qiang Zhou, Fenggui Liu, Lianyou Liu

**Affiliations:** ^1^ State Key Laboratory of Earth Surface Processes and Resource Ecology Beijing Normal University Beijing China; ^2^ Key Laboratory of Environmental Change and Natural Disaster, MOE Beijing Normal University Beijing China; ^3^ Academy of Plateau Science and Sustainability People's Government of Qinghai Province and Beijing Normal University Xining China

**Keywords:** carbon emission, earthquake, earthquake fissures, frozen soil, global climate change, Qinghai‐Tibet plateau

## Abstract

The Qinghai‐Tibet Plateau (QTP) has an extensive frozen soil distribution and intense geological tectonic activity. Our surveys reveal that Qinghai‐Tibet Plateau earthquakes can not only damage infrastructure but also significantly impact carbon dioxide emissions. Fissures created by earthquakes expose deep, frozen soils to the air and, in turn, accelerate soil carbon emissions. We measured average soil carbon emission rates of 968.53 g CO_2_ m^−2^·a^−1^ on the fissure sidewall and 514.79 g CO_2_ m^−2^·a^−1^ at the fissure bottom. We estimated that the total soil carbon emission flux from fissures caused by M ≥ 6.9 earthquakes on the Qinghai‐Tibet Plateau from 326 B.C. to 2022 is 1.83 × 10^12^ g CO_2_ a^−1^; this value is equivalent to 0.51% ~ 1.48% and 2.34% ~ 5.14% of the increased annual average carbon sink resulting from the national ecological restoration projects targeting forest protection and grassland conservation in China, respectively. These earthquake fissures thus increased the soil carbon emission rate by 0.71 g CO_2_ m^−2^·a^−1^ and significantly increased the total carbon emissions. This finding shows that repairing earthquake fissures could play a very important role in coping with global climate change.

## Graphical abstract

Earthquakes on the Qinghai‐Tibet Plateau not only damage infrastructure but also increase carbon dioxide emissions. This study found that fissures caused by these earthquakes expose frozen soils to the air, leading to an increase in soil carbon emission rates by 0.71 g CO_2_ m^−2^·a^−1^. This finding highlights the profound impact of soil carbon emissions from earthquake fissures on global change, contributes to reducing uncertainty in global carbon budgets, and provides scientific guidance for designing carbon neutrality timelines in earthquake‐prone regions.

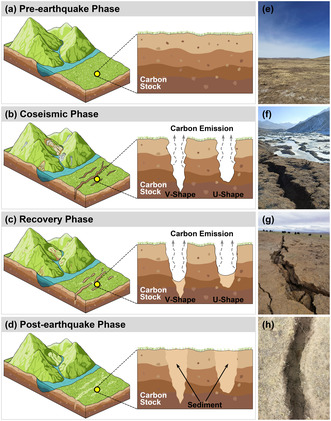

## 1 Introduction

As the “third pole” of the Earth, the Qinghai‐Tibet Plateau is the highest and largest permafrost distribution area in the middle and low latitudes of the world (Cheng et al. 2019). The permafrost area accounts for approximately 40% of the total area of the Qinghai‐Tibet Plateau (Zou et al. 2017). Under low‐temperature environmental conditions, the decomposition rate of soil organic matter in permafrost is low, and a large amount of organic carbon is thus stored in permafrost (Ding et al. 2019; Carvalhais et al. 2014). However, the permafrost on the Qinghai‐Tibet Plateau is very sensitive to global climate change due to the relatively high ground radiation warming, thin thickness, and extremely unstable thermal state of the permafrost in this region (Schädel et al. 2014; Wu et al. 2010). In the context of global warming, the Qinghai Tibet Plateau is warming more prominently than the rest of the world's permafrost regions, and the permafrost here has been significantly degraded, leading to the weakening of the carbon sink capacity of local ecosystems and even the transformation of these ecosystems from carbon sinks to carbon sources (Yao et al. 2018; Mu et al. 2017).

The Qinghai‐Tibet Plateau is also an area with strong tectonic and seismic activity in China (Deng et al. 2014). Over the past two decades, a series of large earthquakes occurred on the Qinghai‐Tibet Plateau, showing a trend of increasing magnitudes and occurrence frequencies (Zhan et al. 2021). Earthquake activity has significant implications for carbon emissions. Previous studies have demonstrated that earthquakes can loosen soils, fracture rocks, and alter subsurface hydrothermal systems, causing the degassing of underground soils and carbonate rocks (Girault et al. 2018; D'Incecco et al. 2021). The carbon dioxide released during the degassing process will quickly migrate into the atmosphere through active faults and hydrothermal systems (Padrón et al. 2008, Liu et al. 2023a). Additionally, earthquake fissures increase the exposure of soil organic matter, accelerating its decomposition. Especially on the Qinghai‐Tibet Plateau, where solar radiation is intense and diurnal temperature variations are significant, earthquake fissures exacerbate the exposure of shallow and deep permafrost in high‐altitude areas. This exposure intensifies soil heat exchange, further accelerating carbon emissions under high‐radiation conditions on the plateau. However, research in this field remains relatively limited.

**FIGURE 1:**
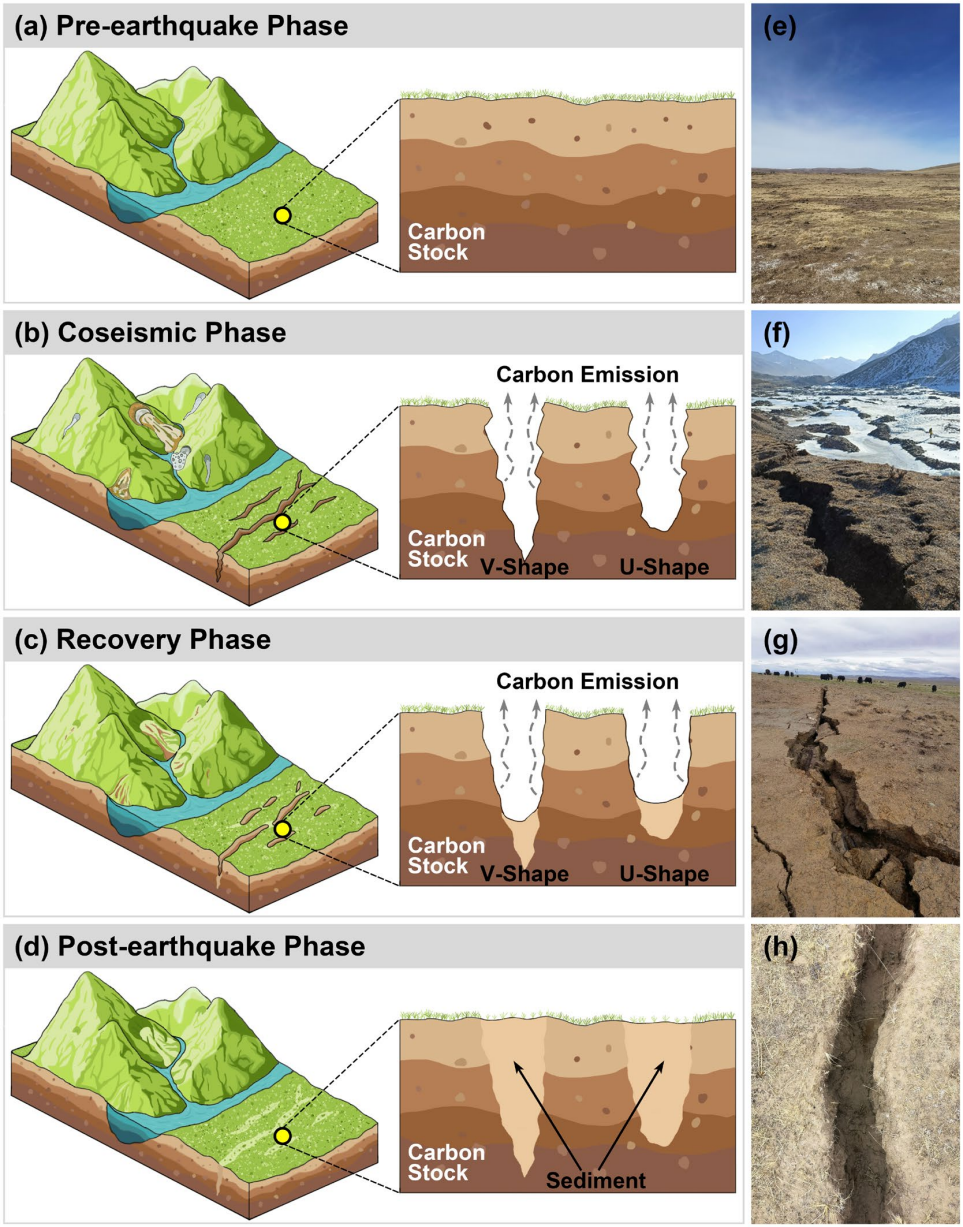
Earthquake fissures accelerate the carbon emissions of soils (including frozen soils). (a) The vertical profile of undisturbed area where organic carbon is stored in the soil. (b) The vertical profile of earthquake fissures showing V‐shaped and U‐shaped fissure and the carbon stocked in the soil being released along the fissures. (c) The vertical profile of fissures after the earthquake and fissures have begun to heal but are still continuing carbon emissions. (d) The vertical profile of earthquake fissures that have been fully recovered and completely filled with sediment. (e) Surface landscape undisturbed by earthquakes. (f) Newly formed earthquake fissures from the Menyuan earthquake. (g) The Maduo earthquake fissure filled with fissure sidewall material. (h) The Maduo earthquake fissure filled with wind deposits.

This study, based on field observations conducted in the high‐intensity zones of the Maduo Ms7.2 earthquake on May 22, 2021, and the Menyuan Ms6.9 earthquake on January 8, 2022, confirms that earthquake fissures significantly expand the exposed soil area on the Qinghai‐Tibet Plateau and accelerate soil carbon emissions. By measuring the length, width, depth, and recovery time of these fissures, an estimation model for soil carbon emission exposure area caused by earthquake fissures was developed. Furthermore, using historical earthquake data on the Qinghai‐Tibet Plateau, the total soil carbon emissions caused by large earthquakes (M ≥ 6.9) from 326 B.C. to 2022 were estimated. This study aims to elucidate the mechanisms by which earthquake fissures expose soils and accelerate carbon emissions (Figure 1), providing a scientific basis for assessing the impacts of earthquakes on regional carbon cycles and global climate change, offering data support for optimizing ecological engineering efforts in earthquake‐prone areas and promoting high‐quality regional development.

## 2 Materials and Methods

### 2.1 Measurement of Soil Carbon Emission From Earthquake Fissures

**FIGURE 2:**
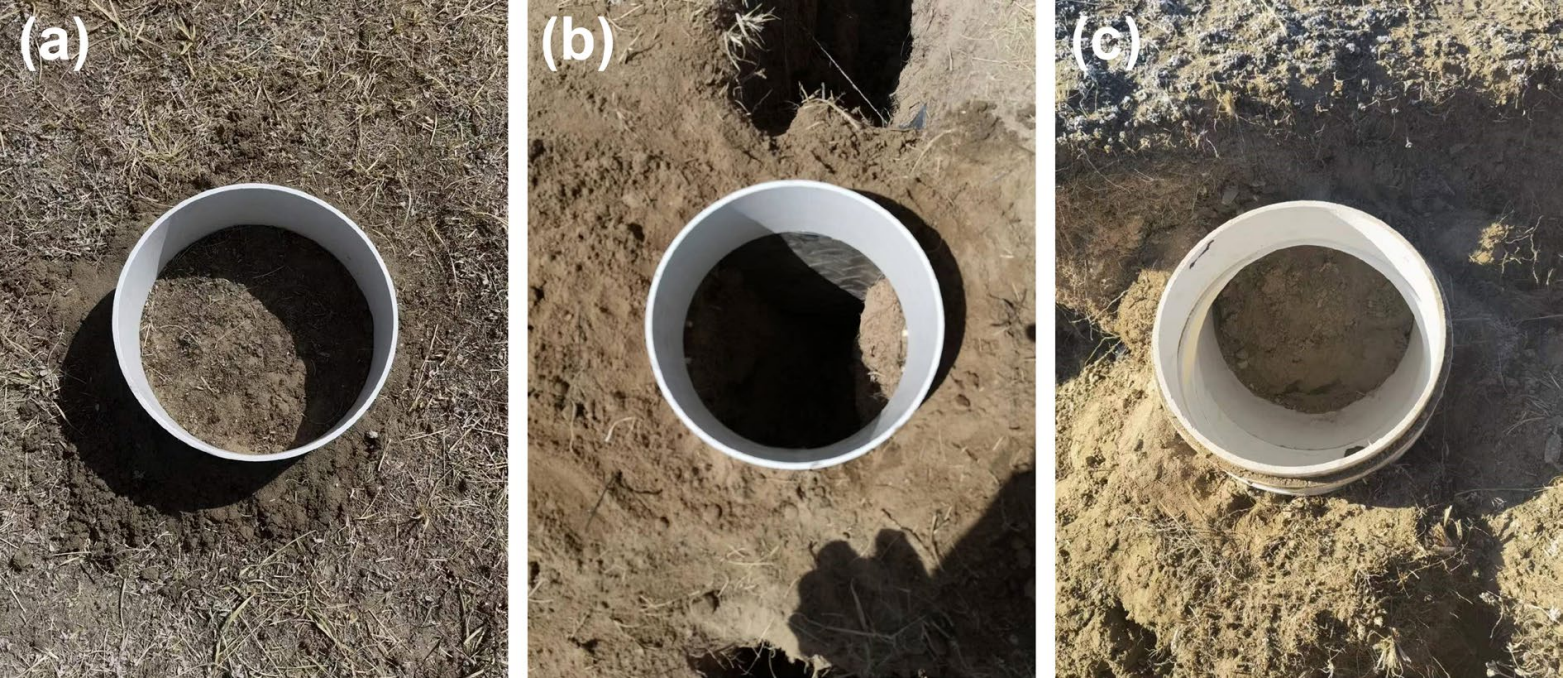
Design of the carbon emission measurements from fissures created in the Maduo earthquake. **(**a) The control group (carbon emissions from a land surface without fissures). (b) The test group (carbon emissions from a fissure with a width of 20 cm and depth of 40 cm). (c) The test group (carbon emission from the fissure sidewall; the fixed base for measuring was a 90° elbow with an inner diameter of 20 cm).

To measure soil carbon emission from earthquake fissures, we conducted field control measurements in high‐intensity areas of the 2021‐05‐22 Maduo Ms7.2 earthquake and 2022‐01‐08 Menyuan Ms6.9 earthquake (Table 1, Figure 2).

**TABLE 1:** Carbon emission results obtained from fissures created during the Maduo earthquake.

Measurement experiment	Earthquake fissure information
Control group	Uncracked surface, no fissures
Test group I	6 cm wide, 20 cm deep
Test group II	6 cm wide, 40 cm deep
Test group III	6 cm wide, 50 cm deep
Test group IV	10 cm wide, 40 cm deep
Test group V	20 cm wide, 40 cm deep
Test group VI	Fissure sidewall
Test group VII	Fissure bottom

#### 2.1.1 Control Group

Areas with similar vegetation and soil environmental conditions near the analyzed earthquake fissures were selected as the control group; at each site, a fixed base with an inner diameter of 20 cm and a height of 8 cm was laid, and the base was buried 3 cm deep. Before the measurements were taken, the vegetation was cut off within the base range, and only the soil respiration was measured.

#### 2.1.2 Test Group

To explore the impact of the widths and depths of earthquake fissures on soil carbon emissions, three groups of fissures with the same depth and different widths and three groups of fissures with the same width and different depths were selected as the test groups. A fixed base with an inner diameter of 20 cm and a height of 8 cm was laid at each fissure. The base was buried 3 cm deep. Two thin iron plates were inserted into the fissure and sealed with soil to ensure that the environment was closed during the measurement process. To measure the soil carbon emissions at the sidewalls and bottoms of the earthquake fissures, a fixed base with an inner diameter of 20 cm and a height of 8 cm was arranged, and the base was buried at a depth of 3 cm. Similar to the control group, the vegetation within the base range was also cut off.

#### 2.1.3 Data Measurement

The soil carbon fluxes were measured every hour from 8:00 to 17:00. Three groups of data were measured each time, and the average value was taken. A total of 855 groups of data were measured, including 530 groups corresponding to the Maduo earthquake and 325 groups corresponding to the Menyuan earthquake.

### 2.2 Total Length of Earthquake Fissures

The estimations were based on the field survey of the Maduo earthquake conducted by Zhou Bao et al. (Zhou et al. 2023). According to the survey data, the Maduo earthquake formed 653 earthquake fissures along five active faults (Table 2).

**TABLE 2:** Maduo earthquake fissure information (Zhou et al. 2023).

Active fault	Number of earthquake fissures	Fissure length range (m)
Maduo‐Gande fault	35	16 ~ 300
Maqu‐Duoqueshan fault	75	280 ~ 2200
Kamuka fault	12	50 ~ 760
Kunlun‐Jiangcuo fault	451	35 ~ 800
Southern edge of Gande fault	80	2 ~ 650

Based on Table 2, the length range ($L_{\mathrm{RM}}^{\prime }$) of the Maduo earthquake fissures was calculated as follows:

(1) $$L_{\mathrm{RM}}^{\prime }=\frac{1}{n}\sum_{n}^{i}L_{i,\min}\sim \frac{1}{n}\sum_{n}^{i}L_{i,\max}$$

where n is the number of active faults investigated in the Maduo earthquake, $L_{i,\min}$ is the minimum length of fissures on the active faults, and $L_{i,\max}$ is the maximum length of the fissures. The length range ($L_{\mathrm{RM}}^{\prime }$) of the Maduo earthquake fissures was calculated as 76.6 ~ 942 m. To assess the maximum carbon emissions of earthquake fissures, the maximum length of 942 m was taken as the length of a single fissure ($L_{M}^{\prime }$) resulting from the Maduo earthquake, and the total length ($L_{M}$) of the Maduo earthquake fissures was calculated as follows:

(2) $$\begin{array}{c} L_{M}=L_{M}^{\prime }\times N_{M} \end{array}$$

where $N_{M}$ is set in reference to the 653 earthquake fissures corresponding to the Maduo earthquake; the total length ($L_{M}$) of fissures resulting from this earthquake was found to be 615,126 m.

At the same time, based on the Chinese earthquake catalog (CENC 2022) containing events that occurred between 1995 and 2022, information about earthquakes with M ≥ 6.9 on the Qinghai‐Tibet Plateau was obtained. The total length of fissures ($L_{k}$) of each earthquake was calculated as follows:

(3) $$\begin{array}{c} L_{k}=\frac{l_{k}}{l_{M}}\times L_{M} \end{array}$$

where$\;L_{k}$ is the total length of fissures of the k^th^ M ≥ 6.9 earthquake, $l_{k}$ is the length of coseismic surface rupture of this earthquake, $l_{M}$ is the length of the coseismic surface rupture of the Maduo earthquake, and $L_{M}$ is the total length of fissures resulting from the Maduo earthquake.

The information on earthquakes with a magnitude of M ≥ 6.9 from 326 B.C. to 2022 was obtained from the historical earthquake catalog of the Qinghai‐Tibet Plateau (Wang 2022). For earthquakes for which it is difficult to obtain the length data of coseismic surface ruptures, the following empirical relationship (Deng and Zhang 1984) can be used to calculate this value:

(4) $$\begin{array}{c} M_{k}=5.92+0.88\log l_{k} \end{array}$$

where $M_{k}$ is the magnitude of the k^th^ M ≥ 6.9 earthquake; $l_{k}$ is the length of the coseismic surface rupture of this earthquake. Then, the total length of the earthquake fissures can be calculated by formula (3).

### 2.3 Average Width of Earthquake Fissures

The empirical relationship between the earthquake magnitude and surface movement can be expressed as follows (Wells and Coppersmith 1994):

(5) $$\begin{array}{c} M_{k}=6.81+0.78\log W_{k} \end{array}$$

where $M_{k}$ is the magnitude of the k^th^ M ≥ 6.9 earthquake and $W_{k}$ is the average width of the resulting earthquake fissures.

### 2.4 Average Depth of Earthquake Fissures

According to the field survey data on the depths of the earthquake fissures in the intensity‐IX area of the Menyuan earthquake, it was found that the maximum depth of earthquake fissures was 1.9 m. Considering that the soil carbon stock on the Qinghai‐Tibet Plateau is mainly concentrated in the soil layer of 0 ~ 3 m and the soil organic carbon stored in the deep soil layer greater than 3 m is very small (Liu et al. 2023b; Yu et al. 2023), this study assumes that the average depth of earthquake fissures on the Qinghai‐Tibet Plateau is 3 m (D=3) and only calculates the earthquake fissures area and soil carbon emissions in this situation.

### 2.5 Time of Earthquake Fissures Recovery

**FIGURE 3:**
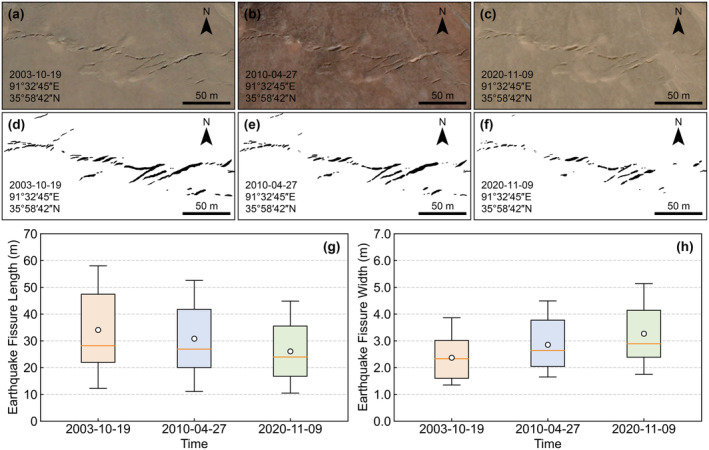
Temporal and spatial variation characteristics of earthquake fissure length and width. (a ~ c) Google Earth remote sensing image of the earthquake fissures in the East Kunlun fault caused by the 2001‐11‐14 Kunlun Ms8.1 earthquake. (d ~ f) Earthquake fissures extracted based on visual interpretation. (g) Temporal variation of earthquake fissure length. (h) Temporal variation of earthquake fissure width.

Considering that the 2001‐11‐14 Kunlun (Qinghai) Ms8.1 earthquake was the largest magnitude earthquake on the Qinghai‐Tibet Plateau from 1995 to 2022, we selected the East Kunlun Fault which was the seismogenic fault of the Kunlun earthquake, as the study subject and we analyzed the temporal variation patterns of the earthquake fissures lengths and widths based on high‐resolution remote sensing images from Google Earth. The results showed that the lengths of the earthquake fissures gradually shortened over time. Meanwhile, material from the fissure sidewalls is deposited at the bottom of the fissures under the influence of gravity, causing the fissure widths to widen (Figure 3). Since remote sensing cannot capture changes in the depth of the fissures, the variation patterns of fissures depth remain unknown for the time being. Statistical results indicate that the decrease rate of earthquake fissure length is 0.49 m·a^−1^ (p<0.05) and the increase rate of earthquake fissure width is 0.05 m·a^−1^ (p<0.05).

### 2.6 Earthquake Fissure Area of a Single Earthquake

The earthquake fissure areas of individual earthquakes were calculated by the following formula:

(6) $$\begin{array}{c} S_{w,k,t}=L_{k,t}\times D\times 2 \end{array}$$

(7) $$\begin{array}{c} S_{b,k,t}=L_{k,t}\times W_{k,t} \end{array}$$

where $S_{w,k,t}$ is the sidewall area in the t^th^ year of the k^th^ M ≥ 6.9 earthquake, $S_{b,k,t}$ is the bottom area in the t^th^ year of the k^th^ M ≥ 6.9 earthquake, $L_{k,t}$ is the earthquake fissure length in the t^th^ year of the k^th^ M ≥ 6.9 earthquake, $W_{k,t}$ is the fissure width in the t^th^ year of the k^th^ M ≥ 6.9 earthquake, and D is the average depth of the M ≥ 6.9 earthquake which is assumed to be 3 m.

### 2.7 Total Area of Earthquake Fissures

The area was calculated using the following formula:

(8) $$\begin{array}{c} S=S_{w}+S_{b}=\int \left(\sum_{n}^{k}S_{w,k,t}+\sum_{n}^{k}S_{b,k,t}\right)\mathrm{dt}/T \end{array}$$

where S is the total area of earthquake fissures on the Qinghai‐Tibet Plateau, $S_{w}$ is the total area of the fissure sidewalls, $S_{b}$ is the total area of the fissure bottoms, n is the number of earthquakes with M ≥ 6.9, $S_{w,k,t}$ is the sidewall area in the t^th^ year of the k^th^ M ≥ 6.9 earthquake, $S_{b,k,t}$ is the bottom area in the t^th^ year of the k^th^ M ≥ 6.9 earthquake, and T is the time since the earthquake occurred. Considering the changes in the fissures over time, S, $S_{w}$, and $S_{b}$ are all multi‐year average results.

### 2.8 Total Carbon Emissions From Earthquake Fissures

The total carbon emissions from fissures were calculated by the following formula:

(9) $$\begin{array}{c} E=S_{w}\times E_{w}+S_{b}\times E_{b} \end{array}$$

where E is the total carbon emissions from fissures on the Qinghai‐Tibet Plateau, $S_{w}$ is the total sidewall area, $E_{w}$ is the average soil carbon emission rate of the fissure sidewalls, $S_{b}$ is the total bottom area, and $E_{b}$ is the average soil carbon emission rate at the fissure bottom.

### 2.9 Calculation Process

**FIGURE 4:**
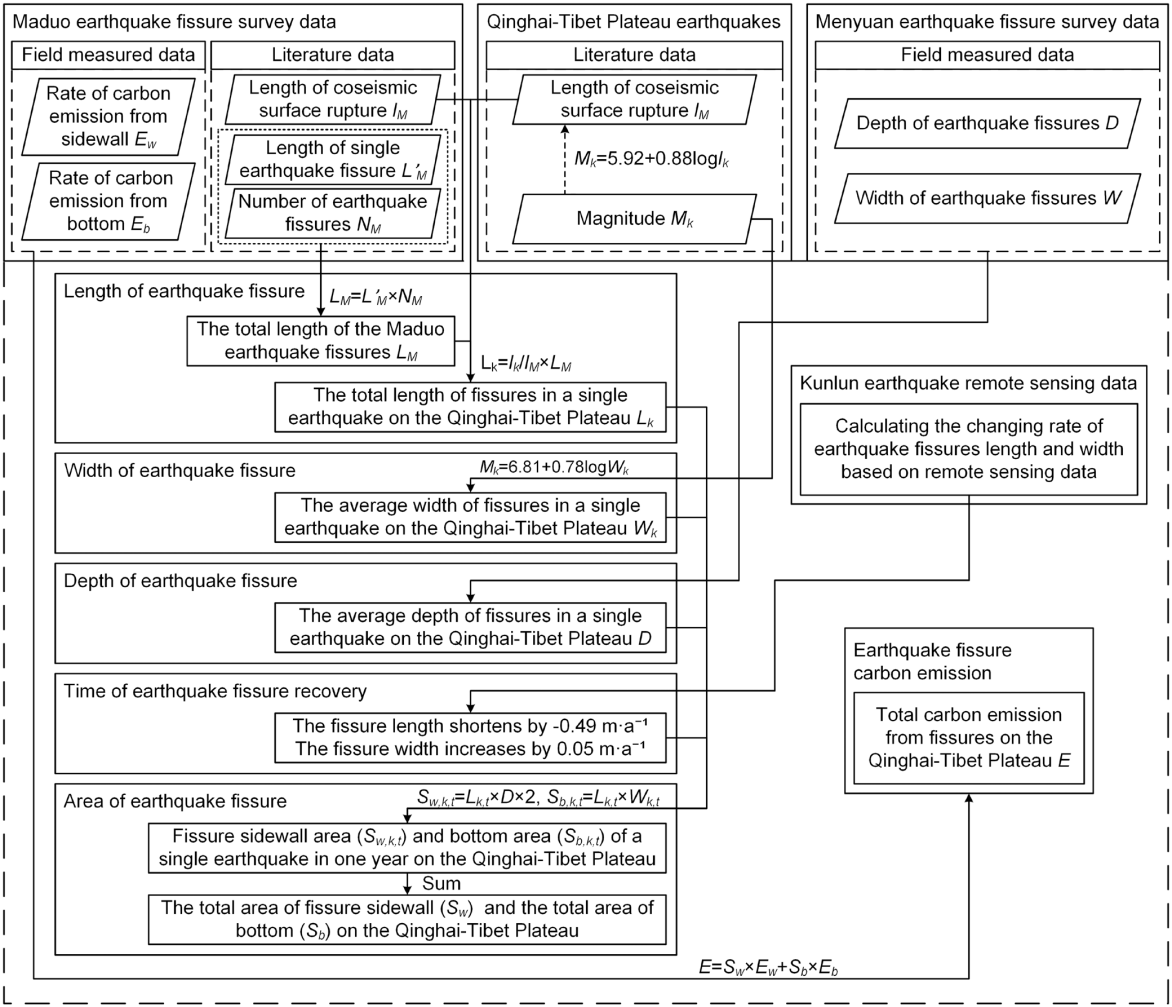
Flowchart showing the calculation process of carbon emissions from earthquake fissures.

The overall calculation process of carbon emissions from earthquake fissures on the Qinghai‐Tibet Plateau can be expressed in the flowchart shown below (Figure 4).

## 3 Result

### 3.1 Soil Carbon Emission From Earthquake Fissures

From April to July 2022, we conducted field‐positioning observations of carbon (CO_2_) emissions at earthquake fissures in the high‐intensity areas of the 2021‐05‐22 Maduo (Qinghai) Ms7.4 earthquake (4200 m) and the 2022‐01‐08 Menyuan (Qinghai) Ms6.9 earthquake (3200 m). By setting up comparative observation experiments, the carbon emissions from earthquake fissures with different widths and depths were determined with a soil carbon flux measurement system (Li‐8100A). On the measurement surface with a diameter of 0.20 m (Table 1, Figure 2), the average soil carbon emission rate of the uncracked surface (vegetation removal) was found to be 1524.95 g CO_2_ m^−2^·a^−1^, and the rate of the fissure sidewall was 968.53 g CO_2_ m^−2^·a^−1^, comprising 63.51% of that of the uncracked surface, an area equivalent to 1.06 times the soil carbon emission rate of 916.78 g CO_2_ m^−2^·a^−1^ caused by the degradation of permafrost on the Qinghai‐Tibet Plateau under the RCP8.5 scenario (Boscha et al. 2017). The average carbon emission rate at the bottom of the fissure was 514.79 g CO_2_ m^−2^·a^−1^, only 33.76% of that measured on the uncracked surface soil.

**FIGURE 5:**
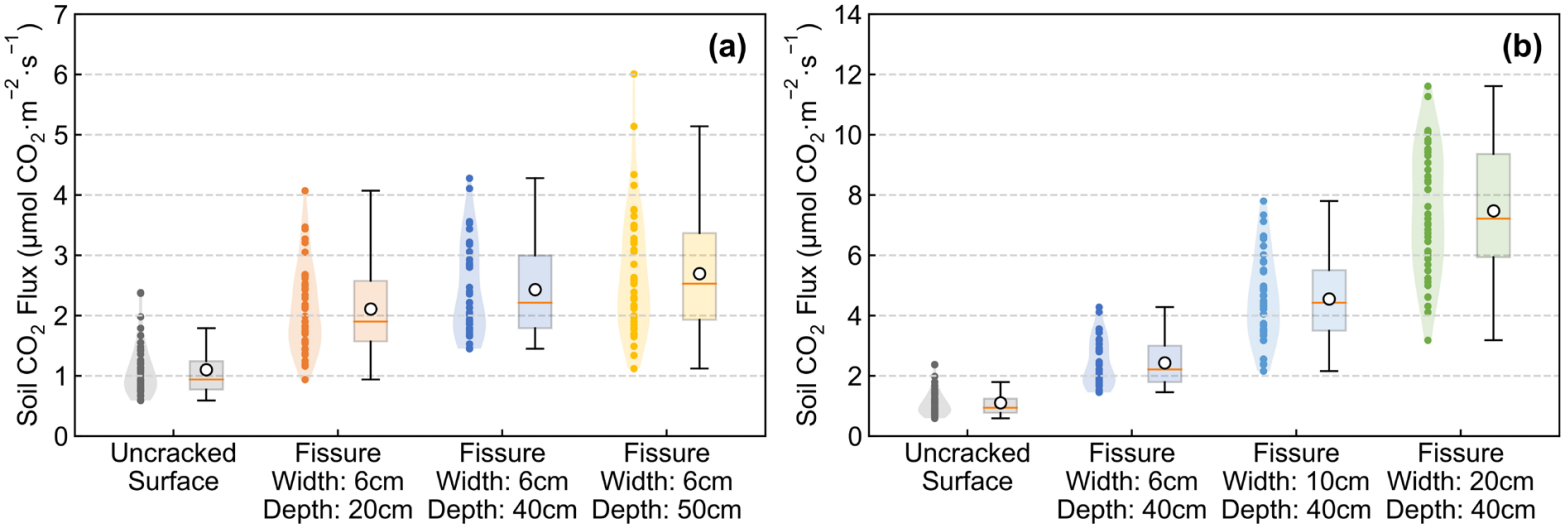
Comparison of soil carbon emissions from earthquake fissures with different depths and widths in high‐intensity areas affected by the Maduo earthquake. (a) The soil carbon emissions from earthquake fissures of the same width and different depths. (b) The soil carbon emissions from earthquake fissures of the same depth and different widths.

The main reason for the measured difference was that surface soils can receive more solar radiation than fissures, resulting in the ground temperature being higher. However, the opening of earthquake fissures increases the surface area over which soil carbon emissions can be released. For a fissure of a certain length, when the fissure becomes deeper and wider, the underground soil exposure area will become larger, thus leading to a significant increase in the total carbon dioxide emissions (Figure 5). Earthquake fissures enable the originally buried underground frozen soils to become in contact with the atmosphere directly, thus greatly enhancing the thermal exchange between the soils and air, accelerating frozen soil degradation, and ultimately increasing soil carbon emissions.

### 3.2 Total Soil Carbon Emission From Earthquake Fissures

**FIGURE 6:**
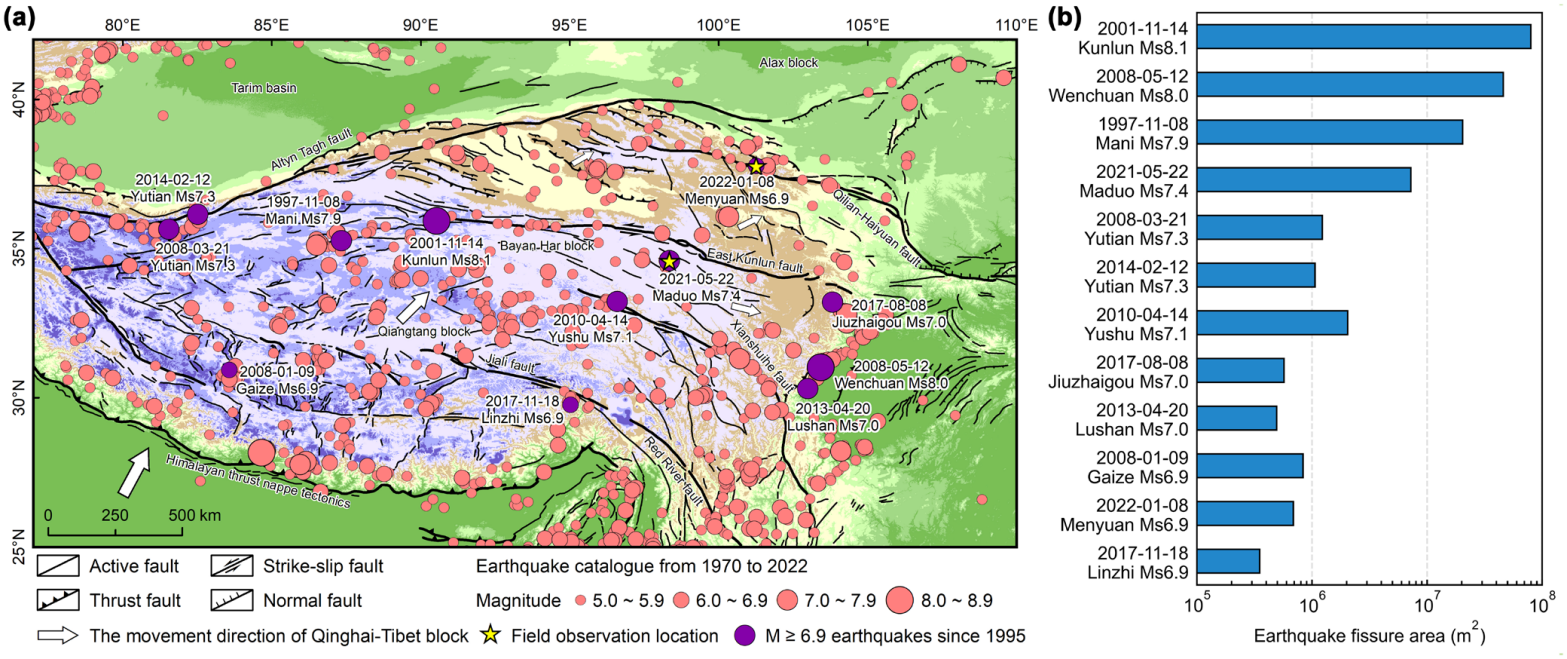
Earthquakes that have occurred on the Qinghai‐Tibet Plateau from 1970 to 2022. (a) The earthquakes (CENC 2022) from 1970 to 2022 and the active fault distribution (Tapponnier et al. 2001) on the Qinghai‐Tibet Plateau. (b) The earthquake fissure area of 12 earthquakes with M ≥ 6.9 occurred on the Qinghai‐Tibet Plateau from 1995 to 2022.

When earthquakes occur, frozen soils are separated on both sides as V‐shaped fissures form a newly increased soil carbon emission area. In the specific estimation process, since the depth and width varied greatly among each earthquake fissure, the areas of the two sidewalls and fissure bottom were measured according to a U‐shaped fissure scheme. From 1995 to 2022, 12 earthquakes with M ≥ 6.9 occurred on the Qinghai‐Tibet Plateau. The area of fissures caused by all earthquakes was calculated (Figure 6).

Referring to the average fissure length of the Maduo earthquake, the newly increased soil carbon emission area caused by the 12 earthquakes on the Qinghai‐Tibet Plateau was estimated to be 1.60 × 10^8^ m^2^; the total area of the fissure sidewalls was 1.32 × 10^8^ m^2^, and the total area of the fissure bottoms was 0.28 × 10^9^ m^2^. By applying formulas (6), (7), and (8), we calculated that the total fissure area of 101 earthquakes with M ≥ 6.9 that have occurred from 326 B.C. to 2022 on the Qinghai‐Tibet Plateau was 1.97 × 10^9^ m^2^; the total area of the fissure sidewalls was 1.80 × 10^9^ m^2^; and the total area of the fissure bottoms was 0.17 × 10^9^ m^2^. The area of the Qinghai‐Tibet Plateau (Zhang, Li, and Zheng 2002) is 2.57 × 10^12^ m^2^. From these findings, the rate of soil carbon emissions from fissures caused by 12 earthquakes with M ≥ 6.9 from 1995 to 2022 increased by 0.06 g CO_2_ m^−2^·a^−1^, and caused by 101 earthquakes with M ≥ 6.9 from 326 B.C. to 2022 increased by 0.71 g CO_2_ m^−2^·a^−1^.

Based on the average soil carbon emission rates of the sidewalls and bottoms of the earthquake fissures, the total soil carbon emissions of the 12 earthquakes were estimated to be 1.42 × 10^11^ g CO_2_·a^−1^. When the 101 earthquakes with M ≥ 6.9 that occurred on the Qinghai‐Tibet Plateau from 326 B.C. to 2022 were included, the total soil carbon emissions from these earthquake fissures were estimated to be 1.83 × 10^12^ g CO_2_·a^−1^. The total soil carbon emissions from fissures were equivalent to 1.00% of the frozen soil annual average carbon emissions caused by permafrost degradation on the Qinghai‐Tibet Plateau under the influence of climate warming (Schuur et al. 2009), 3.53% of the carbon emissions of Qinghai Province in 2019 (CEADs 2022), 0.31% ~ 0.92% of the annual average carbon sink of the net ecosystem on the Qinghai‐Tibet Plateau (Wei et al. 2021), and 0.51% ~ 1.48% and 2.34% ~ 5.14% of the increased annual average carbon sinks corresponding to the national ecological restoration projects targeting forest protection and grassland conservation in China, respectively (Lu et al. 2018).

## 4 Discussion

This study considered the healing time of earthquake fissures by focusing on changes in fissure length and width. However, the variations in fissure length, width, and depth are relatively complex across different geological structures and sedimentary environments. Furthermore, due to the limitations of remote sensing technology, it is currently impossible to estimate changes in earthquake fissure depth. More field observations are necessary to deepen our understanding of the fissure formation and healing processes, as well as the time span involved, in order to more accurately estimate the area of earthquake fissures.

It is important to note that this study mainly focuses on the impact of earthquake fissures on soil organic carbon emissions. Considering that the majority of soil organic carbon on the Qinghai‐Tibet Plateau is concentrated in the soil layer of 0–3 m on the surface (Wang et al. 2020; Liu et al. 2023b; Yu et al. 2023), the average depth of earthquake fissure is used as 3 m for calculating the soil carbon emissions from earthquake fissures. However, the average depth of active fault zones on the Qinghai‐Tibet Plateau is about 20 km (Zhang et al. 2022). The energy released by earthquakes not only causes visible surface fissures but can also result in soil loosening and rock fragmentation, leading to the formation of many smaller fissures underground, which could be several kilometers deep. These inconspicuous earthquake fissures may also contribute to the emission of soil organic carbon, but due to the limitations in instrumentation, we have not yet directly observed them. Additionally, these subtle earthquake fissures are also important pathways for soil and carbonate degassing during earthquakes. Several studies have pointed out that the short‐term soil and rock degassing after earthquakes will quickly release substantial amounts of carbon dioxide (Cui et al. 2017; Girault et al. 2018; Liu et al. 2023a). Therefore, when comprehensively assessing the impact of entire earthquake on carbon emissions, this source of carbon release must also be considered.

The soil carbon storage under different vegetation types and the soil physical and chemical conditions in different sedimentary environments have different effects on the decomposition of organic matter (Li et al. 2021; Wang et al. 2021), which will lead to differences in soil carbon emissions from earthquake fissures in different regions. Considering that the main vegetation types in the Qinghai‐Tibet Plateau are alpine meadows and alpine shrub meadows (Chai et al. 2018), this study selected the Maduo earthquake area (the main vegetation type is alpine meadows) and the Menyuan earthquake area (the main vegetation type is alpine shrub meadows) to observe soil carbon emissions from earthquake fissures. In actual measurements, we also found that under the same soil temperature and soil moisture conditions, the soil carbon emission rate of earthquake fissures in the alpine shrub meadow area is higher than that in the alpine meadow area. It should be pointed out that this study mainly focuses on the permafrost area of the Qinghai‐Tibet Plateau. Due to significant differences in soil carbon emissions under different environments, further research and exploration are needed to estimate soil carbon emissions from earthquake fissures in non‐permafrost regions and to analyze the characteristics of soil carbon emissions from earthquake fissures under different vegetation types and sedimentary environments.

In addition, for the Qinghai‐Tibet Plateau, especially in high‐altitude permafrost regions, the formation of earthquake fissures may have multiple impacts on the carbon cycle. On the one hand, earthquake fissures may penetrate deep permafrost layers, breaking through ice wedges and gradually releasing the carbon dioxide stored within. The estimation results of this study include this part of carbon emissions to a certain extent. On the other hand, the presence of earthquake fissures facilitates the accumulation of meltwater, precipitation, and groundwater, which may promote the formation of ice wedges to some extent. Under the backdrop of global warming, the formation and melting of ice wedges will significantly alter the hydrothermal conditions of permafrost, potentially triggering surface thermo‐erosion collapse, accelerating permafrost degradation, and further increasing carbon emissions (Turetsky et al. 2019; Parmentier et al. 2024). Thus, earthquakes and earthquake fissures may have far‐reaching impacts on the ecologically fragile Qinghai‐Tibet Plateau. These complex ecological effects warrant sustained attention and further investigation through the establishment of long‐term observation systems.

Due to limitations in data acquisition, this study conservatively estimated the impact of earthquake fissures on soil carbon emissions on the Qinghai‐Tibet Plateau. To improve accuracy, further research is needed to improve the earthquake fissure area estimation model, considering factors such as vegetation type, soil type, and environmental conditions that affect soil carbon emissions. At the same time, it is necessary to explore the long‐term effects of earthquakes and earthquake fissures on the ecological environment, establish a long‐term monitoring system for soil carbon emissions after earthquakes, and comprehensively assess the soil carbon emissions caused by earthquake fissures over different time scales. Based on the above discussion, it is possible to estimate the global soil carbon emissions caused by earthquake fissures, reduce the uncertainty in global carbon budgets, and provide scientific guidance for designing carbon neutrality timelines for earthquake‐prone regions around the world.

## 5 Conclusion

In conclusion, the earthquake fissures on the Qinghai‐Tibet Plateau have significantly increased the carbon emission area, accelerated the exchanges of water and heat in the plateau frozen soils, increased the carbon emission rate by 0.71 g CO_2_ m^−2^·a^−1^, and significantly increased the total carbon emissions. These findings indicate that in addition to the ecological restoration of the Qinghai‐Tibet Plateau, earthquake fissure repair should be a focus of stakeholders, as earthquake fissure repair may play an equally important role in realizing the carbon‐neutral strategy and coping with global climate change.

## Author Contributions

**Peijun Shi:** formal analysis, investigation, methodology, supervision, validation, writing – original draft, writing – review and editing. **Xiaokang Hu:** formal analysis, investigation, methodology, validation, visualization, writing – original draft, writing – review and editing. **Heyi Yang:** formal analysis, investigation, validation, visualization, writing – original draft. **Lu Jiang:** investigation, validation, writing – original draft. **Yonggui Ma:** investigation, resources. **Haiping Tang:** methodology, resources, validation. **Qiang Zhou:** resources, validation. **Fenggui Liu:** resources, validation. **Lianyou Liu:** methodology, validation, writing – review and editing.

## Conflicts of Interest

The authors declare no conflicts of interest.

## Data Availability

The field data of carbon emissions is available from Zenodo at https://doi.org/10.5281/zenodo.14533817. The code that supports the findings of this study are openly available in Zenodo at https://doi.org/10.5281/zenodo.14525847.Earthquake event data were obtained from the China Earthquake Networks Center (CENC) at https://doi.org/10.5281/zenodo.14525785 and Wang (2022) at https://doi.org/10.11888/SolidEar.tpdc.272827.
